# Effects of Pre-Curing on the Structure and Properties of Paper-Based Materials

**DOI:** 10.3390/polym15122702

**Published:** 2023-06-16

**Authors:** Mingcen Lin, Wenling Zhou, Ye Yao, Jingxiang Chen, Chunhui Zhang

**Affiliations:** 1 State Key Laboratory of Pulp and Paper Engineering, South China University of Technology, Guangzhou 510640, China; 202010105802@mail.scut.edu.cn (M.L.);; 2 Chinese-German Institute of Engineering, Zhejiang University of Science and Technology, Hangzhou 310023, China

**Keywords:** paper-based material, pre-curing, phenolic resin, mechanical properties

## Abstract

Paper-based friction material is a typical paper-based composite that is usually cured via hot-pressing. This curing method does not account for the effect of pressure on the matrix resin, resulting in uneven distribution of resin in the material and reducing the mechanical properties of friction materials. To overcome the above shortcomings, a pre-curing method was introduced before hot-pressing, and the effects of different pre-curing degrees on the surface morphology and mechanical properties of paper-based friction materials were studied. The pre-curing degree significantly affected the resin distribution and interfacial bonding strength of the paper-based friction material. When the material was cured at 160 °C for 10 min, the pre-curing degree reached 60%. At this point, most of the resin was in a gel state, which could retain abundant pore structures on the material surface without causing mechanical damage to the fiber and resin matrix during hot-pressing. Ultimately, the paper-based friction material exhibited improved static mechanical properties, decreased permanent deformation, and reasonable dynamic mechanical properties.

## 1. Introduction

Paper-based friction material is a new type of paper-based composite that functions in automatic transmission fluid and is comprised of three components: reinforcing fiber, adhesive, and filler [1]. Compared to other wet friction materials, paper-based friction materials have higher porosity and compression resilience, resulting in high and stable friction coefficients, lower wear rates, and leading energy absorption at different pressure and speed levels [2,3]. Resin matrix is the key component affecting the overall strength, friction and wear properties of friction materials. The most common resin matrix is thermosetting cashew shell oil-modified phenolic resin (CMPR), which is also the most widely used adhesive in various wet friction materials [4]. Several studies have indicated that CMPR can effectively improve the friction and wear stability of friction materials due to its toughness and fading resistance [5,6,7].

In previous studies, most researchers discuss the friction and wear mechanism of resin in paper-based friction materials, and some obtained enhanced thermal and mechanical properties using high-performance adhesives [8,9,10,11]. The distribution and cross-linking degree of the resin matrix are important factors affecting the mechanical, friction and wear properties of friction materials [12]. These properties are restricted by various operation methods, such as impregnation, curing, and hot-pressing, during the preparation of paper-based friction materials. The preparation process for traditional paper-based friction materials begins with preparing preformed sheets through papermaking, which are then impregnated with adhesives and finally solidified in a hot press. The uncured resin is easy to squeeze onto the material surface, resulting in uneven distribution of the resin. Specifically, the surface resin content is high in few pores, and low with many others. Eventually, the lubricating oil film formed on the surface of the friction material cannot be effectively drained through the pores, thereby reducing the friction coefficient and heat transfer performance of the material [13,14]. Therefore, exploring a new method for curing resin and optimizing the material preparation process are imperative.

In the field of resin-based composites, research on the curing of resin is relatively mature. Earlier studies indicated that the curing degree of the resin matrix would directly affect its microstructure, which would in turn affect the interfacial bond strength of the composites. Most researchers have predicted or verified the effect of the curing degree of phenolic resin on polymer structures using the reaction mechanism [15,16]. Improper curing processes can lead to unacceptable structural defects in paper-based friction materials, such as residual stress, warping, voids, or other undesirable effects. Zhang et al. explained that the performance of materials is directly related to the curing degree of resins, which affects their viscoelastic behavior and leads to changes in the mechanical properties of materials [17]. In terms of friction materials, Pablo et al. discussed the important influence of resin content on the curing rate [18]. Most literature reported the relationship between the amount of resin and friction performance under different working conditions [1,19]. In addition, other literature compared the performance of various resins and summarized that CMPR has the best friction performance [5,6]. However, few studies have analyzed the curing process for resin and explored the effect of curing degrees on the hot-pressing of paper-based friction materials.

In this study, the curing process and mechanism of CMPR resin were studied by analyzing its thermal properties. Subsequently, the uncured paper-based material was pre-treated using a pre-curing approach under different conditions. The structural changes of paper-based materials before and after hot pressing were observed under optical and electron microscopes. The effects of pre-curing on the mechanical properties and dynamic thermomechanical properties of paper-based materials were explored. Finally, we evaluated whether this experiment could guide the preparation of paper-based friction materials.

## 2. Materials and Methods

### 2.1. Raw Material

The mixture of pitch-based carbon fibers (Kureha Chemical Co., Ltd., Osaka, Japan), Kevlar fibers (Teijin Co., Ltd., Tokyo, Japan), and cotton fibers (Shanghai Hanlun Special Fiber Material Co., Ltd., Shanghai, China) were used as reinforcement. Cashew shell oil-modified phenolic resin (Mitsui Chemicals Co., Ltd., Tokyo, Japan) containing hexamethylenetetramine was used as the adhesive. Polyethylene oxide (standard analytical pure, Sinopharm Chemical Reagent Co., Ltd., Shanghai, China) was used as a dispersant for the mixture of fibers, and industrial ethanol (C_2_H_5_OH, ≥95%, Sinopharm Chemical Reagent Co., Ltd., Shanghai, China) was used as a solvent for phenolic resin powder.

### 2.2. Sample Preparation

**Figure 1:**
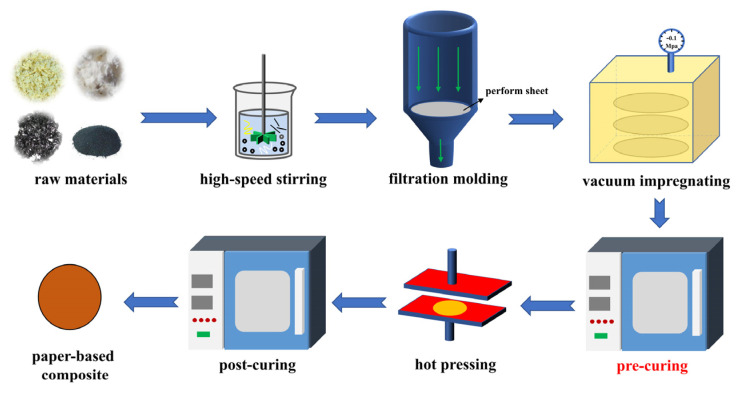
Flowchart of the preparation of paper-based materials.

The three types of fibers are mixed evenly in water containing polyethylene oxide via a high—shear disperser and poured into a paper—forming machine. The mixed liquid was immediately filtered by vacuum to obtain preformed wet sheets, and dried in a ventilated environment. The paper was dissolved in industrial ethanol and impregnated with resin for 15 min in a self-made vacuum dipping box with a vacuum of −0.1 MPa. After infiltration, the preform sheets were air-dried again at room temperature and pre-cured in an oven. Finally, the samples were hot-pressed to 0.5 mm under a pressure of 10 mpa in a hot press at 170 °C and post-cured in an oven at 170 °C. Thus, paper-based materials were obtained. Figure 1 shows the general workflow of paper-based material preparation.

In this experiment, seven kinds of paper-based materials with different pre-curing conditions were prepared, as shown in Table 1. 

### 2.3. Testing Method and Equipment

A differential scanning calorimeter (DSC) 204F1(Netzsch, Bavaria Asia, Germany) was used to analyze the heat reaction during the curing process, and data were obtained from a temperature range of 50–200 °C at a heating rate of 5 °C·min^−1^ under nitrogen atmosphere.

The curing degree of the resin was tested via an extraction method, and the test method was implemented following GB/T 2576-2005. The cured samples were cut into pieces and placed into a filtered paper bag, extracted with ethanol for 16 h, and finally dried and weighed.

The surface morphologies of the samples were observed via scanning electron microscopy (SEM) (Phenom G2 Pro Y, Phenom-World, Eindhoven, Netherlands) and ultra-depth-of-field optical microscopy (Smartzoom 5, Carl Zeiss AG, Oberkochen, Germany), respectively. Gold coating was applied to the sample surface to offer conductivity prior to SEM observation. The changes before and after hot-pressing were compared.

**Figure 2:**
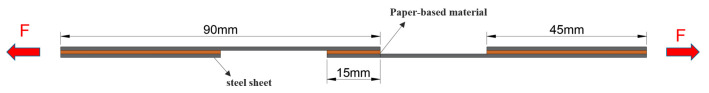
Diagram of the shear strength test.

The shear, compression and recovery tests of the samples under dry conditions at room temperature were carried out on an electronic universal testing machine (Instron 5565, Instron Engineering Corporation, MA, USA). The shear strength test jigs are shown in Figure 2. The dimensions of the shearing sample are 15 mm×15 mm×0.5 mm. The two sides of the sample were bonded to the steel sheet with resin adhesive to ensure that it does not fall off during the shearing process. The jig distance was controlled to 75 mm, and the stretch speed was controlled to 0.5 mm/min until the sample failed in shear. The experimental results were expressed as the arithmetic mean of the shear modulus.

**Figure 3:**
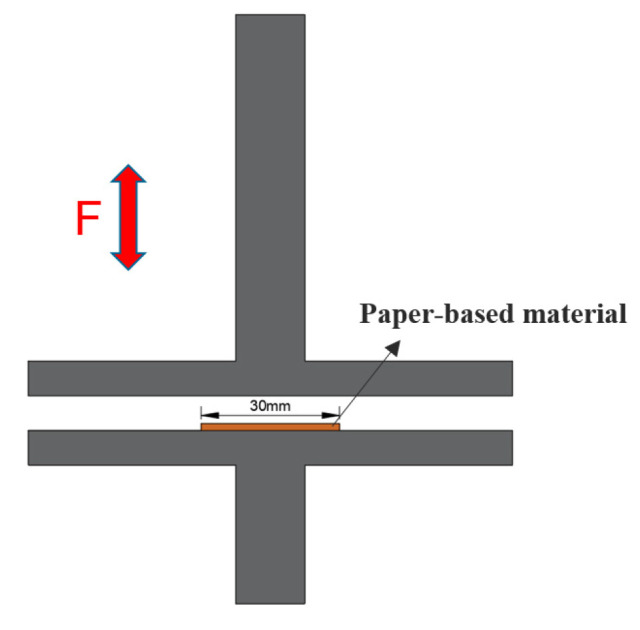
Diagram of the compression and recovery test.

The compression and recovery test jig is shown in Figure 3. The dimensions of the compression and recovery sample is 30 mm × 30 mm × 0.5 mm. First, the preload of 90 N (0.1 MPa) was applied to the sample. Subsequently, the compression block was uniformly loaded at 100 N/s until the load was 4500 N (5 MPa) and was unloaded to 90 N at the same rate. The above operation was repeated ten times. The permanent set rate was calculated as the total deformation of 10 test cycles. The compressibility (ψ) and recovery (ξ) were calculated as follows:

(1) $$\psi =\frac{S_{1}-S_{0}}{h}\times 100\%\;$$

(2) $$\xi =\frac{S_{1}-S_{2}}{S_{1}-S_{0}}\times 100\%$$

where *h* is the thickness of samples under preload, mm; *S*_0_ is the displacement under preload, mm. *S*_1_ is the displacement under maximum load, mm; *S*_2_ is the displacement back to the preload, mm.

A dynamic mechanical analyzer (DMA) (TAQ800, TA Instruments, DE, USA) was used to test the viscoelasticity of the samples. The storage and loss moduli changes of the materials were measured using a tensile jig maintaining an oscillation frequency of 5 Hz and an amplitude of 15 μm in all tests.

All measurements were taken three times, and the mean value was used.

## 3. Results and Discussion

### 3.1. Curing Reactions of CMPR

**Figure 4:**
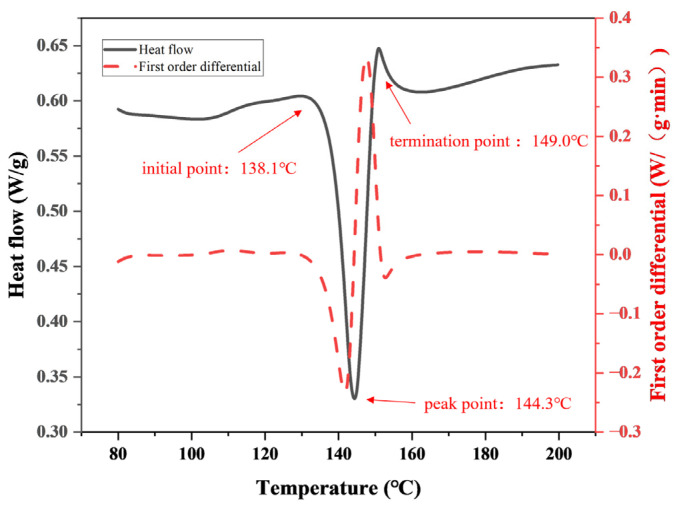
DSC profiles of the CMPR at a heating rate of 5 °C·min^−1^.

To study the curing reaction of a resin matrix, the curing behavior of a CMPR at a heating rate of 5 °C/min was monitored via DSC. The results presented in Figure 4 show that the cashew-modified phenolic resin had only one curing exothermic peak as the temperature increased. The initial curing temperature, the exothermic peak temperature, and the termination curing temperature of the CMPR were 138.1 °C, 144.3 °C, and 149.0 °C, respectively.

The curing behavior indicated that the initial exothermic temperature was the lowest curing temperature of the modified phenolic resin. At this point, the reaction rate between groups occurred more quickly; nevertheless, the collision rate between macromolecules was slow, and the reaction process was gentle [20]. As the temperature increased, the multifunctional monomer of the polymer began to cross-link and gel, leading to a sharp increase in viscosity. Therefore, the cross-linking rate was the largest at the peak of the exothermic reaction. When the temperature was further increased, the degree of cross-linking in the system became remarkably large, and the movement of active groups was inhibited. The curing reaction rate was reduced and tended to be stable after the endpoint exothermic temperature. The resin was assumed to undergo the two stages of gelation (from liquid to a rubbery state) and vitrification (from a rubbery state to a glassy state) during this period [21]. Thus, this curing process had an important influence on the mechanical properties of the composites.

### 3.2. Effects of Pre-Curing Conditions on the Pre-Curing Degree of Materials

In accordance with the curing characteristics of the CMPR, the paper-based materials were pre-cured at different temperatures and times, as shown in Table 1, and the curing degree of the materials was tested. The curing degree is a significant target to measure, in order to characterize the degree of resin cross-linking, which plays an essential role in monitoring the properties of composites. A higher curing degree indicates a higher molecular weight of the monomer that has been cross-linked at a certain time [22].

**Figure 5:**
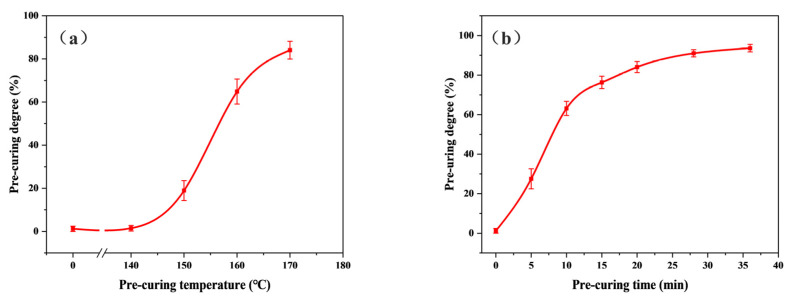
Relationship between pre-curing conditions and pre-curing degree: (**a**) relationship between pre-curing temperature and pre-curing degree in 20 min, (**b**) relationship between pre-curing time and pre-curing degree at 170 °C.

The change in the curing degree of the CMPR with pre-curing times and temperatures is shown in Figure 5. Figure 5a shows that under the same pre-curing time, the relational curve between the pre-curing degree and the pre-curing temperature was “S” type. The trend was in line with the three stages of phenolic resin curing. With increasing pre-curing temperature, the pre-curing degree of the material increased rapidly until the curing degree reached 60%. At this point, more than half of the resin was converted to a glass state, the molecular activity decreased, and the viscosity of the system increased. Macromolecular reactions were hindered, resulting in slower subsequent cross-linking reactions [23].

Figure 5b shows that with increasing pre-curing time, the pre-curing degree of the sample increased rapidly at first, but the growth rate decreased after the pre-curing degree reached 60%. When the reaction time reached 38 min, the pre-curing degree was above 98%. Hence, the resin could be cured rapidly at 170 °C, but the curing reaction of the resin was inhibited when the pre-curing degree reached 60%. The resin was almost completely cross-linked into a network structure after 38 min.

### 3.3. Effects of Pre-Curing on the Morphology of Materials before and after Hot-Pressing

**Figure 6:**
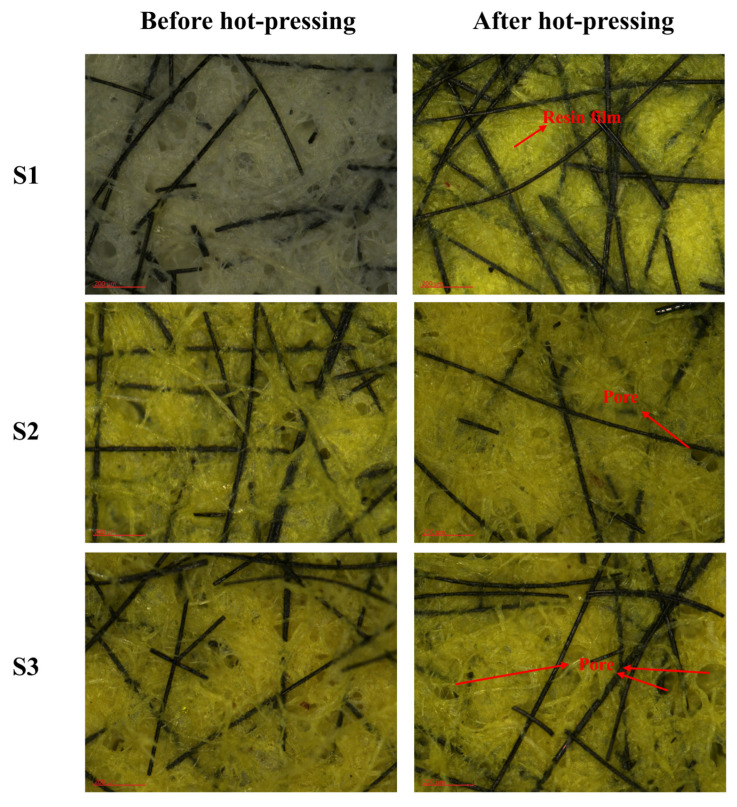

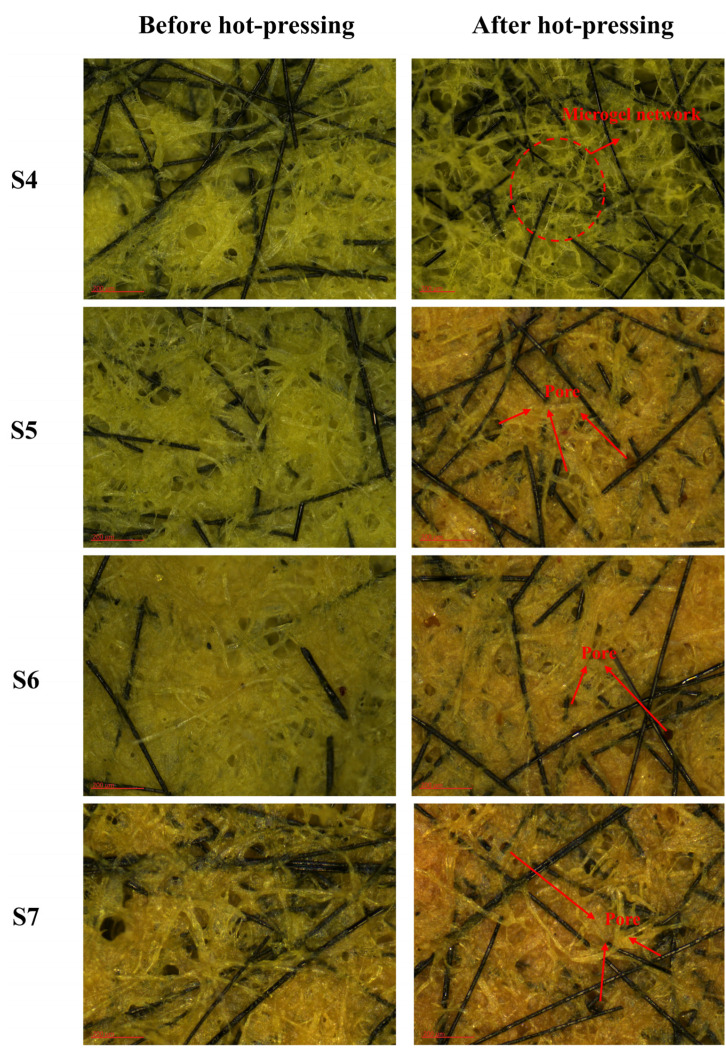
Optical microscopy images of S1–S7 before and after hot-pressing.

**Figure 7:**
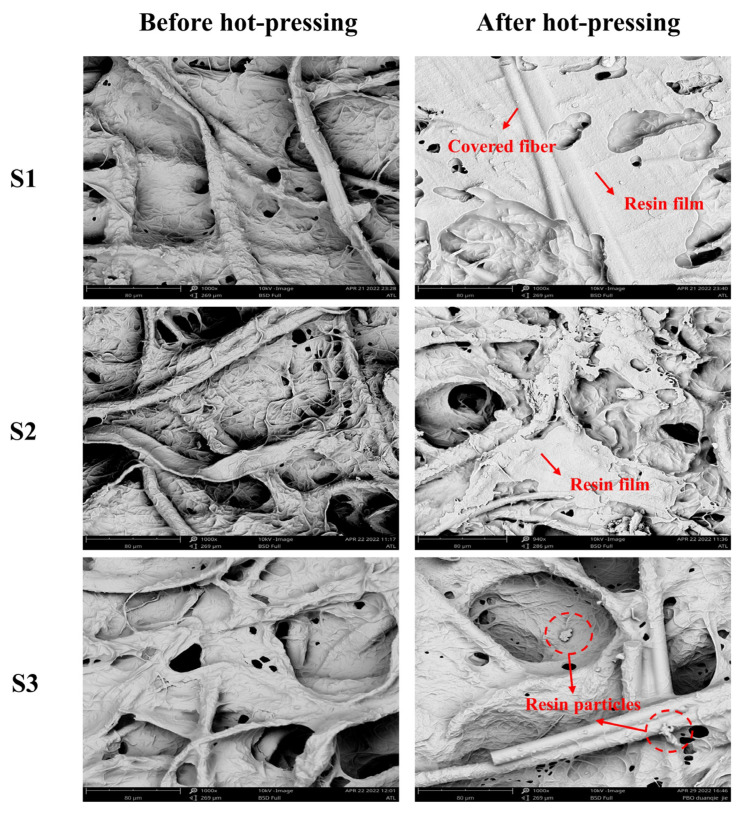

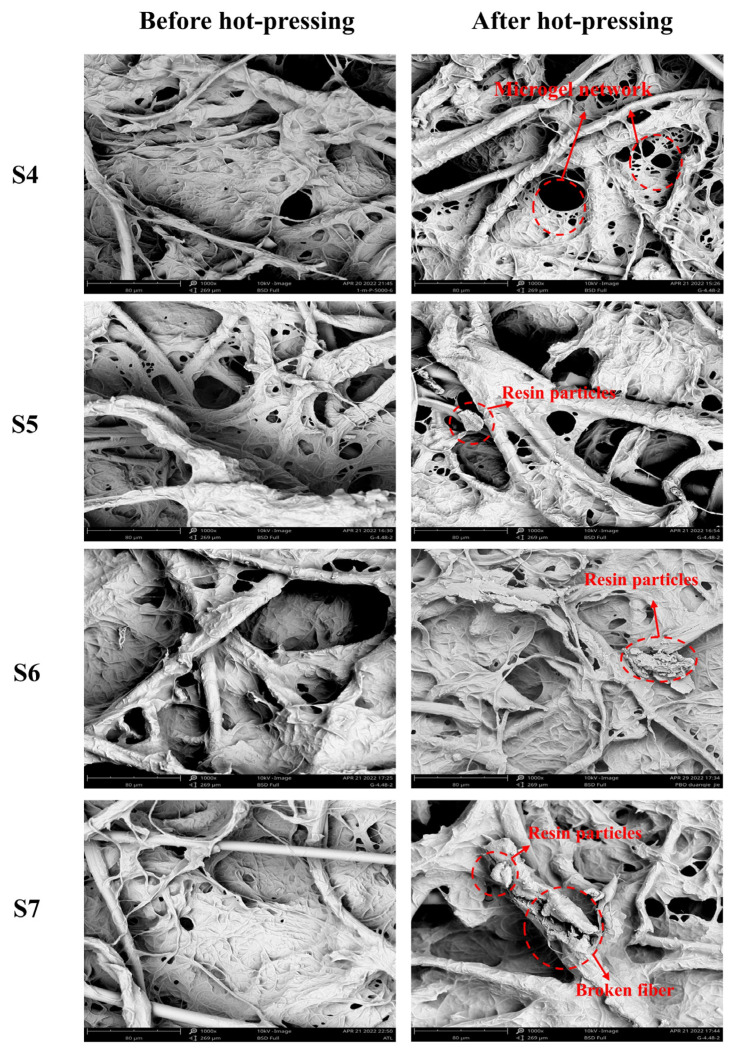
SEM images of S1–S7 before and after hot-pressing.

To investigate the effects of pre-curing on the surface morphology of materials, the surface morphology of the material before and after hot-pressing was characterized using an ultra-depth optical microscope and a scanning electron microscope, as shown in Figure 6 and Figure 7.

The results indicated that most of the resin with good fluidity in sample S1 was extruded to the material surface via hot-pressing, and a layer of resin film was formed, which covered the fiber and filler. The friction performance of paper-based friction materials is attributed to carbon fibers serving as smooth friction films under high contact loads, while the production of resin films hinders the production of carbon fiber friction films [24]. In the application of sample S1, the friction force could not be effectively transferred to the reinforcing fiber, which affected the wear resistance of the material. Meanwhile, less surface pores in the material would hinder the circulation of lubricating oil and reduce the heat resistance of the material [25]. With increasing pre-curing temperatures, the number of pores on the surface of sample S2 after hot-pressing was increased, but there was still a small amount of resin film on the surface.

Sample S4 was further gelled under hot-pressing and formed a microgel network because its pre-curing degree was low, and the resin continued the unfinished gel reaction during the hot-pressing process. The surfaces of samples S3 and S7 showed an abundant pore structure, indicating that hot-pressing exerted a minimal effect on the surface structure and the fluidity of resin of the composites under this pre-curing condition, which was beneficial to the uniform distribution of the resin.

More pores were retained on the surfaces of samples S5–S7, demonstrating that with the increase in pre-curing time, the pre-curing degree of composites increased rapidly, and the effect of hot-pressing on the pore structure of materials decreased.

The SEM images in Figure 7 demonstrate that hot-pressing caused mechanical damage to the sample with the highest pre-curing degree. For samples S3, S5, S6, and S7, the pressure crushed the brittle cured resin and formed resin particles. In particular, the hot-pressing caused the most serious damage to sample S7, where a significant amount of damaged fibers resin fragments were found. In the process of pre-curing, the larger the degree of cross-linking of the resin, the greater the bonding strength with the fiber [26]. When the pressure was transferred, the fiber wrapped by the resin was peeled off from the resin and destroyed. Therefore, the mechanical damage in this process has a great impact on the compressive and shear properties of fiber composites.

Before hot-pressing, the resin was able to evenly penetrate the paper-based materials, which have a good pore structure. However, the hot-pressing process could cause changes in the pore structure of the material and may cause damage to fibers and resins. Using pre-curing and changing the flow state of the resin during hot-pressing could preserve the original pore distribution and reduce the damage of each component, thereby reducing the negative effects caused by hot-pressing.

### 3.4. Effects of Pre-Curing on the Mechanical Properties of Materials

During the working process of the wet clutch, the friction pair relied on the pressure of the piston to force the friction plate into contact with the dual steel plate. The paper-based friction material was compressed and recovered by the change in longitudinal pressure and subjected to shear force to generate torque [27]. Therefore, the compression resilience and shear properties of paper-based friction materials are the two key factors affecting their mechanical properties.

**Figure 8:**
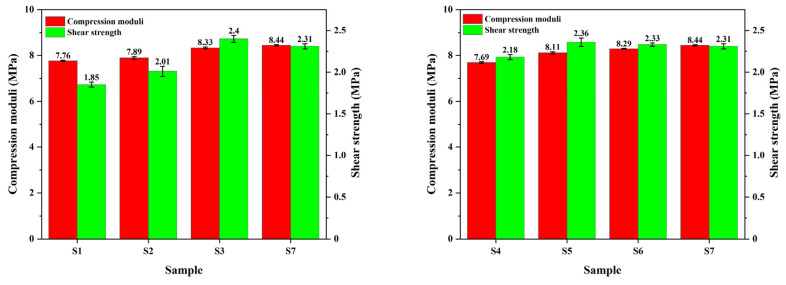
Compression modulus and shear modulus of different paper-based materials.

Figure 8 shows the changes in the compression modulus and shear strength of paper-based materials under different pre-curing conditions. The results indicated that the compression modulus of the materials increased alongside increasing pre-curing temperatures and times. The compression modulus of the composite depended on the compression modulus of the phenolic resin matrix. Therefore, a larger curing degree represented greater rigidity and a higher compression modulus of the composite, which was beneficial for improving the compression resistance of the material. However, the improvement of material rigidity also reduces the engagement stability of materials [28].

In addition, the shear strength indirectly reflects the longitudinal defects of the material and the interfacial bonding strength between the resin and the fiber [29]. The shear strength of the materials first increased and then decreased with increasing pre-curing degrees. The shear moduli of samples S3 and S5 were the highest, which were 2.4 and 2.36 MPa, respectively. When the pre-curing degree was low (such as those for S1 and S2), there was more resin on the material surface than inside, resulting in less bonding between the fiber and the resin matrix and poor shear performance. When the pre-curing degree was high (such as those for S6 and S7), hot-pressing destroyed the high-viscosity resin and produced more defects, which were the direct causes of the decrease in the shear strength of the material. Samples S3 and S5 exhibited the best shear properties, indicating that the hot-pressing caused less damage to the material and that the interface bonding strength was higher under this pre-curing condition. At this point, transferring the load between the fiber and the resin, reducing the pressure concentration, and enhancing the damage resistance of the material were beneficial.

**Figure 9:**
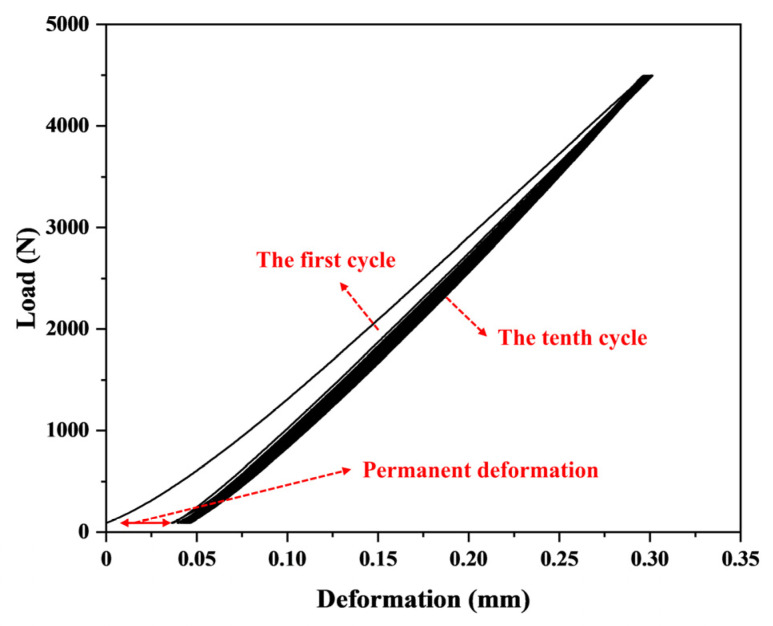
Compression and recovery test of paper-based materials.

The compression and recovery properties of paper-based friction materials are significant factors affecting the bonding stability and thermal stability of friction pairs [30]. Figure 9 shows the stress–strain curve of a paper-based material under 10 compression cycles. The compressibility of the first paper-based material was higher, and the recovery was smaller. With increasing compression cycles, the compressibility decreased, and the recovery increased. Given that the paper-based material had abundant pore structures, the hole wall buckled during the first compression, resulting in the collapse of the hole wall and contact together. The compression deformation was large, and the crushed pores led to a small recovery and permanent deformation. As the number of cycles increased, the deformation of the material tended to be stable, and its compression resilience depended on the elasticity of the undamaged fiber and resin.

**Figure 10:**
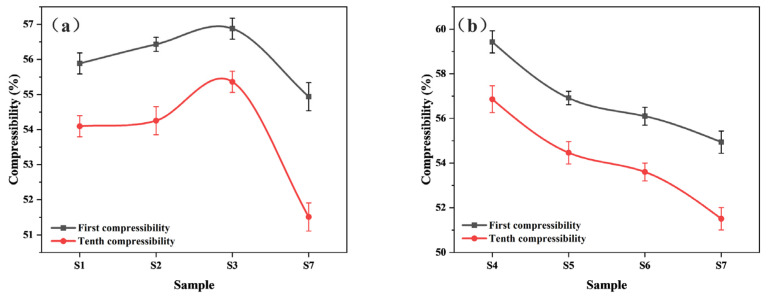
Compressibility of different paper-based materials: (**a**) under different pre-curing temperatures; (**b**) under different pre-curing times.

Higher compressibility and recovery could promote the absorption and discharge of lubricating oil, complete the convective heat transfer with oil, and increase the heat resistance of the material [31]. Figure 10 shows the change in compressibility of paper-based materials under different pre-curing conditions. Figure 10a shows that with increasing pre-curing temperature, the compressibility of the composite first increased and then decreased. When the pre-curing temperature reached 160 °C (sample S3), the compressibility of the first and tenth composite were the maximum, which were 56.88% and 55.36%, respectively. Figure 6 and Figure 7 show that the surface of sample S3 after hot-pressing retained more pores and no fiber damage occurred. The elasticity of the resin and fiber were retained to the greatest extent, which directly resulted in S3 maintaining high compressibility. By contrast, S7 had more fiber and resin damage, resulting in a rapid decrease in the compressibility of the composite. As shown in Figure 10b, with increasing pre-curing time at 170 °C, the compressibility of the material decreased, indicating that the mechanical behavior of the resin matrix was more sensitive at this temperature. The cross-linking was much quicker, resulting in a rapid increase in the modulus of the material and an increase in the amount of damage caused by hot-pressing.

**Figure 11:**
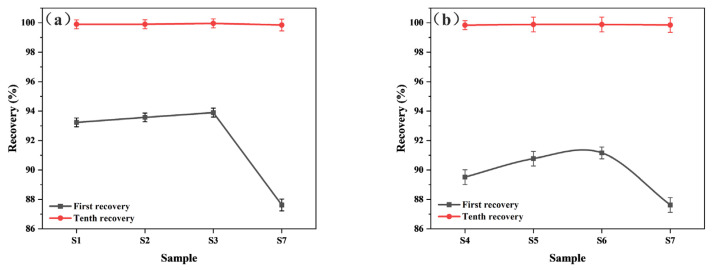
Recovery of different paper-based materials: (**a**) under different pre-curing temperatures; (**b**) under different pre-curing times.

Figure 11 shows the recovery of paper-based materials under different pre-curing conditions. The first recovery of each sample was lower than the tenth recovery, which was due to the irreversible deformation caused by the first pressure. The tenth recovery of each sample was close to 100% quality (no damage occurred) and tended to be stable, so the first recovery of the materials may better reflect the recovery performance.

Figure 11 also shows that the first recovery of the samples increased slowly and then decreased suddenly with an increase in either pre-curing temperature or time. As the pre-curing degree increased, the degree of cross-linking between the resin matrix in the material intensified and the effect of hot-pressing on the distribution of the resin decreased, resulting in an increase in resilience. However, the recovery of sample S7 decreased because the fiber and the resin was destroyed. In addition, the interfacial bonding strength between resin and fiber is also an important factor affecting the material’s recovery performance. We will further analyze the interfacial bonding performance of the material through DMA testing in an upcoming study. Samples S1–S3 had better first recovery than samples S4–S6, indicating that pre-curing under low-temperature conditions could increase those composites’ resilience.

**Figure 12:**
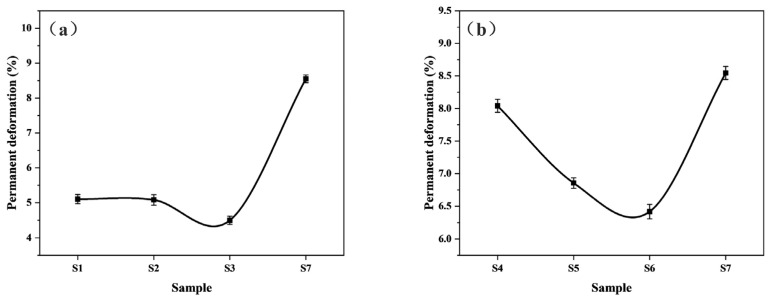
Permanent deformations of different paper-based materials: (**a**) under different pre-curing temperatures; (**b**) under different pre-curing times.

A lower number of permanent deformations could ensure the bonding stability of the friction pair for efficient and stable transmission of torque. Figure 12 shows the changes in the number of permanent deformations of paper-based materials under different pre-curing conditions, which was an intuitive manifestation of the first compression deformation loss. Naturally, the trend corresponded to the first compressibility and recovery of the composite material. Samples S1–S3 exhibited smaller permanent deformations than samples S4–S6. The hot-pressing caused less damage to the material and was more conducive to exerting the mechanical properties of each component in the composite at the pre-curing temperatures of 140–160 °C. In designing the friction material, the friction material has smaller permanent deformations while having higher stable compressibility and recovery to ensure the stability of the clutch engagement.

### 3.5. Effects of Pre-Curing on the Dynamic Mechanical Properties of Materials

**Figure 13:**
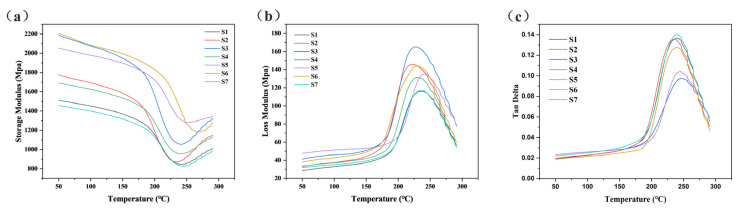
Dynamic mechanical properties of different paper-based materials: (**a**) storage modulus of samples; (**b**) loss modulus of samples; (**c**) loss factors of samples.

The paper-based friction material is a viscoelastic material, and dynamic mechanical analysis is an important method used to study viscoelasticity [32]. Figure 13 shows the dynamic mechanical properties of paper-based materials under different pre-curing conditions.

Figure 13a shows the storage modulus curve, which can reflect the rigidity and deformation recovery ability of the sample and show the bonding strength between the fiber and the interface [28]. The storage modulus of the sample first decreased and then increased with the increase in temperature. Samples S3, S5, and S6 demonstrated higher storage modulus, indicating that they retained the bonding degree between the resin matrix and the fiber to the greatest extent during hot-press curing. On the contrary, the storage modulus of S1, S2, and S4 were low because of their low pre-curing degree and uneven distribution of resin, so the overall bonding strength of fiber and resin were low. Sample S7 had the lowest storage modulus because of the decrease in interfacial bonding strength caused by the failure of its fibers and matrix. This further explains the decrease of sample S7 shear strength, which is closely related to the decrease of its interfacial bonding strength. Figure 13b shows the loss modulus curve, which reflects the viscosity change of the material and the ability to dissipate mechanical energy through internal molecular migration. The trend was opposite to the storage modulus.

The loss factor curve (Figure 13c) describes the ratio of loss modulus to storage modulus, and the peak temperature is the glass transition temperature of the material [28]. The loss factor peaks of samples S3 and S5 were smaller. The bonding strength between fiber and resin in sample S5 was larger, and the energy dissipated by intramolecular friction was smaller under dynamic force. At this point, the structure of the material was more suitable for its mechanical properties during wet clutch engagement. When the material is subjected to force, more pressure is applied to its deformation, while less pressure is dissipated in the form of heat. In addition, the glass transition temperatures of S3 and S5 were 20–30 °C higher than those of the other samples. The glass transition reaction refers to the movement of molecular chain segments; it showed that the resin distribution was uniform and discontinuous in samples S3 and S5. Most of them had strong interfacial bonding with the fiber, and the movement of the molecular chain was weaker. Therefore, both samples showed strong dynamic mechanical properties, corresponding to their surface morphology and mechanical properties. The dynamic mechanical properties of sample S6 were not as effective as its mechanical properties because of the damaged resins and fibers, which led to more interface debonding in high-frequency and high-temperature mechanical tests.

In the field of friction materials, the degree of pre-curing of resin determines the flow state of resin during the hot-pressing process. Hot-pressing can cause damage to paper thermosetting resin-based composites, especially the interfacial bonding strength, which greatly limits the mechanical properties of the material. The pre-curing method in this article can guide the process of optimizing of friction materials reinforced with different resin matrices to reduce the negative effects caused by hot-pressing.

## 4. Conclusions

To overcome the disadvantages of traditional curing approaches for paper-based friction materials and improve their pore structure and mechanical properties, a pre-curing method was studied, which created favorable basic conditions for the post-processing of the paper-based friction materials. Before hot-pressing, the pre-curing degree of the resin exerted an important influence on the resin distribution and pore structure in the material.

Through changing the pre-curing conditions, the curing degree of the material could be controlled. With increasing pre-curing time and temperature, the pre-curing degree of the material increased. When the pre-curing degree of paper-based materials was less than 20%, hot-pressing would squeeze the resin to the surface, resulting in uneven distribution of the resin and blocked pores on the surface. When the pre-curing degree reached 60%, most of the resin system was in a gel state and glass state, which retained the original structure and could be further cured during the hot-pressing process. At this point, the obtained paper-based material was uniform and porous, with excellent static mechanical properties and dynamic mechanical properties at high temperatures. When the pre-curing degree was over 70%, the hot-pressing caused damage to the fibers and resins in the material with larger modulus, resulting in interface defects. With the application of force in the compression and shear directions, the fibers were debonded, which weakened the comprehensive mechanical properties. Given the appropriate pre-curing degree, the mechanical properties of paper-based materials could be significantly improved by pre-curing at 160 °C for 20 min.

## Figures and Tables

**Table 1:** Pre-curing conditions for paper-based materials.

Samples	Pre-Curing Temperature/°C	Pre-Curing Time/min
S1	140	20
S2	150	20
S3	160	20
S4	170	5
S5	170	10
S6	170	15
S7	170	20

## Data Availability

Data will be made available on request.
